# Genomics and transcriptomics of *Xanthomonas campestris* species challenge the concept of core type III effectome

**DOI:** 10.1186/s12864-015-2190-0

**Published:** 2015-11-18

**Authors:** Brice Roux, Stéphanie Bolot, Endrick Guy, Nicolas Denancé, Martine Lautier, Marie-Françoise Jardinaud, Marion Fischer-Le Saux, Perrine Portier, Marie-Agnès Jacques, Lionel Gagnevin, Olivier Pruvost, Emmanuelle Lauber, Matthieu Arlat, Sébastien Carrère, Ralf Koebnik, Laurent D. Noël

**Affiliations:** INRA, UMR 441 Laboratoire des Interactions Plantes Micro-organismes (LIPM), F-31326 Castanet-Tolosan, France; CNRS, UMR 2594 Laboratoire des Interactions Plantes Micro-organismes (LIPM), F-31326 Castanet-Tolosan, France; Université de Toulouse, Université Paul Sabatier, F-31062 Toulouse, France; INRA, UMR 1345 Institut de Recherche en Horticulture et Semences (IRHS), F-49070 Beaucouzé, France; CIRAD, UMR Peuplements Végétaux et Bioagresseurs en Milieu Tropical (PVBMT), F-97410 Saint-Pierre, La Réunion France; Institut de Recherche pour le Développement (IRD), UMR IRD-CIRAD-UM2 Interactions Plantes-Microorganismes-Environnement (IPME), F-34394 Montpellier, Cedex 5 France; Present address: INRA, UMR Institut de Génétique, Environnement et Protection des Plantes, F-35653 Le Rheu, France; INPT-Université de TOULOUSE, ENSAT, Avenue de l’Agrobiopole, Auzeville-Tolosane, 31326 Castanet-Tolosan, France; Present address: INRA, UMR 1345 Institut de Recherche en Horticulture et Semences (IRHS), F-49070, Beaucouzé, France

**Keywords:** *Xanthomonas campestris*, Type III secretion, Effector, *Xanthomonas* outer protein, RNA sequencing, Transcriptome

## Abstract

**Background:**

The bacterial species *Xanthomonas campestris* infects a wide range of *Brassicaceae*. Specific pathovars of this species cause black rot (pv. *campestris*), bacterial blight of stock (pv. *incanae*) or bacterial leaf spot (pv. *raphani*).

**Results:**

In this study, we extended the genomic coverage of the species by sequencing and annotating the genomes of strains from pathovar *incanae* (CFBP 1606R and CFBP 2527R), pathovar *raphani* (CFBP 5828R) and a pathovar formerly named *barbareae* (CFBP 5825R). While comparative analyses identified a large core ORFeome at the species level, the core type III effectome was limited to only three putative type III effectors (XopP, XopF1 and XopAL1). In *Xanthomonas*, these effector proteins are injected inside the plant cells by the type III secretion system and contribute collectively to virulence. A deep and strand-specific RNA sequencing strategy was adopted in order to experimentally refine genome annotation for strain CFBP 5828R. This approach also allowed the experimental definition of novel ORFs and non-coding RNA transcripts. Using a constitutively active allele of *hrpG*, a master regulator of the type III secretion system, a HrpG-dependent regulon of 141 genes co-regulated with the type III secretion system was identified. Importantly, all these genes but seven are positively regulated by HrpG and 56 of those encode components of the Hrp type III secretion system and putative effector proteins.

**Conclusions:**

This dataset is an important resource to mine for novel type III effector proteins as well as for bacterial genes which could contribute to pathogenicity of *X. campestris*.

**Electronic supplementary material:**

The online version of this article (doi:10.1186/s12864-015-2190-0) contains supplementary material, which is available to authorized users.

## Background

Gram-negative bacteria of the species *Xanthomonas campestris* are able to cause disease on *Brassicaceae* and are responsible for important yield and quality losses in brassica crops such as cabbage, radish, cauliflower or Chinese cabbage [1]. Interestingly, *Xanthomonas campestris* isolates are natural pathogens of the model plant species *Arabidopsis thaliana* [2]. Based on host range, mode of infection and the disease symptoms caused on plants, the species was further divided in three pathovars [3]. The pathovar *campestris* regroups strains which are able to cause black rot on at least one cruciferous species. Strains of the pathovar *incanae* cause bacterial blight on garden stock and wallflower while those of pathovar *raphani* are the causal agent of bacterial leaf spot on both cruciferous and solanaceous plants [3]. Finally, weakly or non-pathogenic (NP) strains, which were originally assigned to the pathovars *barbareae* or *armoraciae*, were included into a fourth group of less-defined strains due to their relatedness by multilocus sequence analysis (MLSA) [3, 4]. While strains of pathovars *campestris* and *incanae* use the hydathodes (and wounds) to initiate a vascular infection of the plant, strains of the pathovar *raphani* seem to preferentially use stomata (and wounds) to enter the leaf and colonize the mesophyll.

Extensive molecular genetics of *Xanthomonas campestris* has identified key pathogenicity determinants [5–7] such as extracellular polysaccharides, lipo-polysaccharides, DSF-dependent quorum sensing, extracellular enzymes (proteases, cellulases,…) exported by the type II secretion systems or proteins secreted by the type III secretion (T3S) system. Type III-secreted proteins (T3SP) include type III effector (T3E) proteins which are injected inside the plant cells where many of them interfere with cell physiology and plant immunity. Because the Hrp (hypersensitive response and pathogenicity) T3S system is essential for *Xanthomonas* virulence, it is assumed that the type III effectome is also globally essential for any interaction with the plant. Yet, individual effectors usually have limited or non-significant contribution to pathogenicity when studied alone, probably reflecting functional redundancy and/or additivity between effectors. *hrp* genes are not expressed in *Xanthomonas* cultivated in rich media. Yet, *hrp* gene expression can be induced in *Xanthomonas* in specific minimal media (XVM2, MME, MMX) that were thought to mimic *in planta* conditions [8–10]. Two master regulators of the *hrp* systems which are both required for virulence and *hrp* gene expression in minimal medium have been identified: HrpX is an AraC-type transcriptional activator inducing the expression of all *hrp* operons but *hrpA* upon binding to the plant-inducible promoter (PIP) box (TTCGB-N_15_-TTCGB; B represents C, G, or T) in the promoter region [11, 12]. Expression of most T3E genes is under the control of *hrpX* [13–15]. HrpG, an OmpR-type transcriptional regulator and its putative cognate sensor kinase HpaS control the expression of *hrpX,* the *hrpA* operon and other genes [16, 17]. Interestingly, several point mutations in *hrpG* (hrpG*) can render its activity constitutive in the absence of inducing condition and result in an increased aggressiveness on plants [18].

To date, four complete genome sequences are available in the *X. campestris* species (pathovar *campestris*: 8004, ATCC33913, B100; pathovar *raphani*: 756C) and six draft genomes (pathovar *campestris*: Xca5, JX, B-1459, CN14, CN15 and CN16) [7, 19–25]. These strains have in common a ca. 5-Mb circular chromosome with approx. 65 % G + C content. The presence of plasmids has only been reported in strains B-1459, CN14, CN15 and CN16 [23, 25]. No genomic information is available yet on the pathovar *incanae* and the non-pathogenic group of *X. campestris*. Comparative genomics has proven to be an important tool to mine for pathogenicity determinants, host specificity factors and in particular for T3E [21]. Due to the lack of conserved secretion/translocation signals in T3SP, those can only be predicted by indirect means: *in silico* proteomes can be studied by homology searches with known T3E sequences, by searching for eukaryotic signatures (nuclear localization signals, myristoylation/palmitoylation signals, F-box motifs,…) and/or predicted genes can be scrutinized for the presence of a PIP box. Indeed, co-expression of T3SP with the Hrp T3S system is the rule and has proven to be a powerful tool for the identification of novel T3E in *Xanthomonas* [13], *Ralstonia solanacearum* [26] and *Pseudomonas syringae* [27]. To this end, the analysis of *Xanthomonas* transcriptomes in *hrp*-inducing conditions or in deregulated mutants expressing constitutively the *hrp* regulon has been a major source for the discovery of novel effectors [13–15, 28].

Here, we report on the genome sequencing of four *X. campestris* strains of the pathovars *incanae*, *raphani* and NP and their comparison with publically available *X. campestris* genomes. This approach identified a very limited core type III effectome for this species of *Xanthomonas*. A transcriptomic analysis of the *hrpG* regulon has also been performed in a *X. campestris* pv. *raphani* strain CFBP 5828R and provides important information for the structural annotation of the genes and precious hints for the identification of candidate type III effector proteins as well as genes which could contribute to pathogenicity of *X. campestris*.

## Results

### Genome sequencing and properties

Four *X. campestris* strains belonging to pathovars not or poorly characterized at the genomic levels were selected for this study (Table 1). Two strains of the pathovar *incanae*, CFBP 2527 and CFBP 1606, were chosen because these strains were isolated on two distinct continents at 24 years interval. Notably, strain CFBP 2527, which was isolated from hoary stock (*Matthiola incana*) in the USA in 1950, is the pathotype strain of pathovar *incanae*. Strain CFBP 5828 belongs to the *raphani* pathovar and was isolated from radish in the USA. So far, only one complete genome has been determined in this pathovar [21]. The sequenced strain 756C was isolated from cabbage in East Asia and was classified in the *raphani* pathovar based on its MLSA profile and pathogenicity [3, 29]. Strain CFBP 5825 was initially classified as pathovar *barbareae* and designated as the pathotype strain of this pathovar but has recently been assigned to the *X. campestris* NP (“non-pathogenic”) clade [3].

**Table 1:** Origin of *Xanthomonas campestris* strains and genome properties

Strain	Pathovar^b^	Isolation^b^		Nb of reads^c^	Contigs						Pseudochromosome		
		Host plant	Country and year		Assembly size (bp)	Coverage	Nb contigs^d^	N50	Average size (bp)	Largest (bp)	Nb contigs organised	Size (bp)^e^	GC %
CFBP 5825R	NP^a^	*Barbarea vulgaris*	USA, 1939	PE = 10,697,622	5,053,608	696	8	2,773,827	631,701	2,773,827	7	5,053,372	65.1
				MP = 13,325,195									
CFBP 1606R	*incanae*	*Matthiola incana*	France, 1974	PE = 19,497,157	4,966,388	1057	9	3,800,062	551,820	3,800,062	9	4,967,188	65.2
				MP = 12,873,343									
CFBP 2527R	*incanae*	*Matthiola incana*	USA, 1950	PE = 18,139,985	4,925,175	1142	6	2,485,103	820,862	2,485,103	6	4,925,675	65.1
				MP = 19,259,657									
CFBP 5828R	*raphani*	*Raphanus sativus*	USA, nd	PE = 19,555,502	4,911,085	1029	11	1,210,840	446,462	1,728,667	10	4,911,500	65.4
				MP = 10,836,933									

^a^Non-pathogenic; received as pv. *barbareae*

^b^Properties of the original wild strains (rifampicin sensitive)

^c^PE: paired end (101 bp); MP: mate pair (51 bp)

^d^Larger than 200 bp

^e^with 100-bp gaps between contigs

Shotgun sequencing of genomic DNA of rifampicin-resistant derivatives of these strains (“R” suffix; e.g. CFBP 5825R) was performed on HiSeq2000 Illumina platform. A combination of paired-end (101-bp reads) and mate-pair libraries (51-bp reads) was used reaching 696- to 1142-fold theoretical coverage (Table 1). Genome assembly was performed using a combination of SOAPdenovo [30] and Velvet [31] assemblers and yielded 6 to 11 contigs per genome. These contigs were further ordered into a large pseudochromosome based on the chromosomal organization of *X. campestris* pv. *campestris* strain 8004. Resulting pseudochromosome sizes have both genome sizes (ca. 5Mb) and G + C contents (ca. 65 %) which are typical for *Xanthomonas*. For both strains CFBP 5825R and CFBP 5828R, one small (<1kb) contig showing no homology to *Xanthomonas* chromosomal sequences could not be assembled into the final pseudochromosome. Consistently, we did not detect any endogenous plasmids in those strains (data not shown) nor plasmid-like sequences in their genomes. Due to their highly repetitive nature, transcription activator-like (TAL) effector sequences present in strains CFBP 5825R and CFBP 1606R could not be assembled and are not represented in the final assemblies. Southern blot analyses of *Bam*HI digested genomic DNA indicate that at least one TAL gene is present in each genome (Denancé and Noël, unpublished results). With less than ten scaffolds per genome [GenBank:ATNN00000000, GenBank: ATNO00000000, GenBank: ATNP00000000, GenBank: ATNQ00000000], these draft genomes are sufficiently well assembled to allow gene discovery and gene functional analyses and to develop molecular typing tools.

### Genome annotation and *X. campestris* genomic properties

De novo annotation was performed using Eugene-P (Table 2, https://lipm-browsers.toulouse.inra.fr/pub/xanthomix/): 4447 to 4698 genes, 4262 to 4510 CDS, three ribosomal RNA operons, 53 to 54 tRNA and 125 to 130 other non-coding (nc) RNAs could be identified per genome. ncRNA were predicted using rfam_scan v1.0.2 with Rfam v10.0 based on their homologies to known ncRNA sequences.

**Table 2:** Gene content in the genomes of the newly sequenced *Xanthomonas campestris* strains

Strain	Pathovar	All genes	mRNA^a^	rRNA^b^	tRNA^c^	Other ncRNA^d^
CFBP 5825R	NP^e^	4698	4510	3	54	131
CFBP 1606R	*incanae*	4574	4393	3	53	125
CFBP 2527R	*incanae*	4588	4404	3	54	127
CFBP 5828R	*raphani*	4447	4262	3	53	130
CFBP 5828R + RNAseq^f^	*raphani*	4610	4279	3	53	275

^a^Protein coding sequence

^b^Ribosomal RNA

^c^Transfer RNA

^d^Non coding RNA

^e^Non-pathogenic; received as pv. *barbareae*

^f^Genes predicted using both genomic and RNA sequencing (RNAseq) datasets

**Fig. 1:**
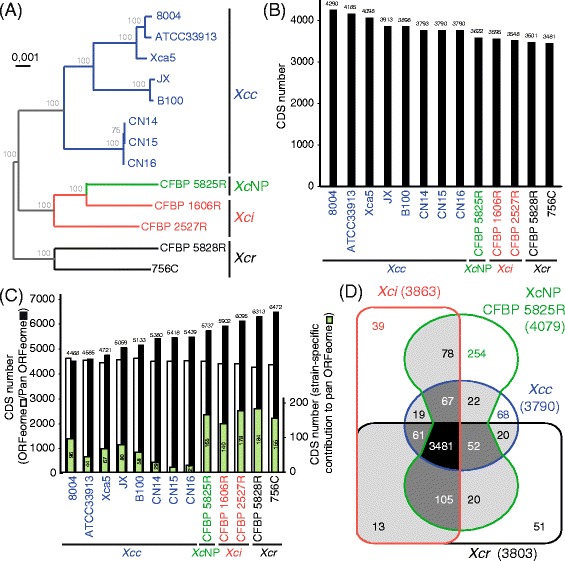
Comparison of 13 publically available and newly sequenced *Xanthomonas campestris* proteomes*.* Orthologous proteins were determined using OrthoMCL software using homogenously re-annotated genomes. **a** A phylogenetic tree of *X. campestris* core proteomes (3481 orthologous coding sequences, CDS) was generated using the PhyML software (Default parameters). Bootstrap values are indicated in grey for each branch. **b** Size of *X. campestris* core ORFeome was determined considering only CDS with a single ortholog per genome. **c** Numbers above black bars indicate the size of the pan ORFeome. Only one CDS per orthology group was considered. The number of annotated CDS per genome is indicated (open bar). The number of isolate-specific CDS is given (green bars). **d** Venn diagram illustrating the number of coding sequences shared among the core ORFeomes (as defined in (b)) in the four *X. campestris* pathovars. Numbers in brackets indicate the number of genes in the core genome of the pathovars. *Xcc* (blue): *X. campestris* pv. *campestris*, *Xci* (red): *X. campestris* pv. *incanae, Xcr* (black): *X. campestris* pv. *raphani*, *Xc*NP (green): *X. campestris* non-pathogenic

In order to determine the phylogenetic relationship of the newly sequenced strains in relation to nine published *X. campestris* genomes [7, 19–24], all genomes were first structurally re-annotated using Eugene-P. The resulting proteomes were compared using OrthoMCL and yielded a *X. campestris* core genome composed of more than 3481 CDS which was used to perform phylogenetic analyses. As inferred from amplified fragment length polymorphism (AFLP) analyses [32], *X. campestris* pv. *campestris*, is organized into at least three clades: Clade XccA contains strains B100 and JX, clade XccC contains strains 8004, Xca5 and ATCC33913 and clade G contains strains CN14, CN15 and CN16 (Fig. 1a). In contrast to strains of pathovar *campestris*, strains of pathovars *incanae* and *raphani* did not cluster in monophyletic groups: this phylogenetic analysis could not fully discriminate between *X. campestris* NP and *X. campestris* pv. *incanae* (Fig. 1a). The phylogenetic relationships at the subspecies level can also be determined using CRISPR systems [33]. CRIPR loci are bacterial immune systems which store fossil records of exogenous DNA acquired during evolution upon phage infection or plasmid acquisition in the so-called spacer regions. Interestingly, *X. raphani* strains CFBP 5828R and 756C were the only strains carrying a CRISPR locus. These two loci harbored identical repeats and 101 and 85 spacers in strains CFBP 5828R and 756C, respectively. The lack of common spacers between these two CRISPR loci and the significant distance separating the core genomes of these two strains (Fig. 1a) support the hypothesis that both strains diverged a long time ago.

The *X. campestris* core ORFeome was composed of 3481 protein coding genes as estimated using OrthoMCL (Fig. 1b and d). In particular, the *X. campestris* pv*. campestris* core ORFeome was composed of 3790 CDS. While each genome contains ca. 4400 CDS, the pan genomes of *X. campestris* and *X. campestris* pv. *campestris* encompass 6472 and 5439 CDS, respectively (Fig. 1c). For *X. campestris* pv. *campestris,* the number of strain-specific CDS in the pan ORFeome was low indicating that these strains are genetically closely related. In contrast, each strains of the other *X. campestris* pathovars contributed more than 140 CDS to the *X. campestris* pan ORFeome probably as a result of the lower number of representative genomes per pathovar available for analysis. These results are in agreement with the phylogenetic relationships observed in the *X. campestris* core genome (Fig. 1a) indicating that the acquisition/fixation of these accessory genes is likely the result of speciation events that were essentially vertically inherited. The core ORFeomes of these four *X. campestris* pathovars were compared (Fig. 1d). These analyses highlighted that the core ORFeomes of pathovars *raphani* and *incanae* are slightly bigger in size than that of pathovar *campestris* which might reflect the lower number of genomes analysed for pathovars *raphani* and *incanae*. The *X. campestris* NP clade was represented by a single strain CFBP 5825R which artificially increased the size of its core ORFeome to 4079 CDS. The identification of these core and pan ORFeome for the species and the different pathovars is an important resource to classify new strains and to mine for determinants of pathogenicity and host specificity.

### Avoidance of FLS2- and EFR-mediated pattern-triggered immunity (PTI) is restricted to pathovar *campestris*

**Fig. 2:**
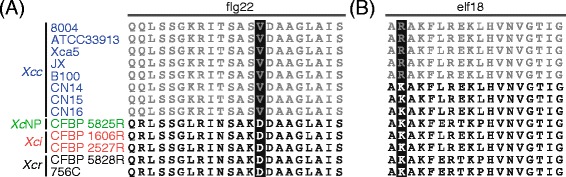
Conservation in *Xanthomonas campestris* of flg22 (**a**) and elf18 (**b**) peptides from FliC and EF-Tu PAMP proteins, respectively. Peptides with/without predicted elicitor activity are in black/grey, respectively. Residues which are essential for elicitor activity and polymorphic in *X. campestris* are underlined in black

Perception of pathogen-associated molecular patterns (PAMP) elicits pattern-triggered immunity which strongly restricts microbial pathogenicity. In this study, we investigated the diversity of two major bacterial PAMP proteins: the flg22 peptide from the FliC flagellin protein which is perceived by the FLS2 (flagellin sensitive) plant immune receptor of Arabidopsis and the elf18 peptide from the elongation factor Tu (EF-Tu) which is monitored by the *Brassicaceae*-specific EF-Tu receptor (EFR). The lack of recognition of the FliC flagellin of *X. campestris* pv. campestris by FLS2 was previously reported [32, 34] and holds true for the eight strains studied here (Fig. 2a). In contrast, analysis of FliC diversity in other pathovars showed that each other FliC isoforms were predicted to be recognized by FLS2. The perception of *X. campestris* elongation factor Tu (EF-Tu) by EFR is probably not systematic either. Though essential residues K4, F5 and R7 of the elicitor peptide elf18 are conserved at the species level, five strains of the pathovar *campestris* (8004, ATCC33913, Xca5, JX and B100; clades XccA and XccC) express an EF-Tu with a K2R substitution (Fig. 2b). This polymorphism likely prevents recognition by EFR since elf18-K2R peptides failed to elicit EFR-dependent responses [35]. These results suggest that avoidance of FLS2- mediated PTI have been acquired first during the evolution of the pathovar *campestris* for FliC and later for EF-Tu.

### A reduced core type III secretome in *X. campestris*

In order to determine the type III secretome of the thirteen *X. campestris* strains, genomes were analyzed manually using tblastn against T3SP reported for the *Xanthomonas* genus ([http://www.xanthomonas.org/t3e.html] and [36]) (Table 3). Presence of genes encoding homologues of T3SP was validated if sequence identity was higher than 60% over the full-length protein. At least 13 T3SP were present for CFBP 5828R, 18 for CFBP 5825R, 21 for CFBP 1606R, and 24 (plus one pseudogene) for CFBP 2527R. These predicted secretomes are rather small, especially considering that they include HrpW, XopA and HpaA which are likely involved in the secretion/translocation process per se. In contrast, the secretome of *X. campestris* pv*. campestris* comprised 22 T3SP on average (min 17, max 27) [32]. AvrXccA1 was not considered in these effectomes because there is no experimental evidence to support its secretion by the T3S system nor its co-regulation with the T3S system.

**Table 3:** Candidate type III-secreted proteins identified in *X. campestris* strains sequenced in this study

Name	Related proteins or synonyms	*XcNP* CFBP5825R^a^	*Xci* CFBP1606R^b^	*Xci* CFBP2527R^c^	*Xcr* CFBP5828R^d^
Candidate type III effectors					
AvrBs2	-	00540	00930	43790	-^e^
AvrXccA2	-	-	-	-	19470
Hax	TAL, Pth, AvrBs3	44610	44690	-	-
XopB	HopD1	-	-	40770	-
XopD1	-	-	12980		-
XopD2				31530	
XopE2	AvrXccE1, HopX	-	-	02540	-
XopE3	AvrXacE2, HopX	-	21880	-	-
XopF1	Hpa4	32150	31370	12940	31070
XopG	HopH, HopAP	09520	09650	34800	-
XopK	-	13060	12950	31570	-
XopL	XopLR	-	-	-	42660
XopN	HopAU1	02730	02740	41710	-
XopP	Hlk	31830	31700	12620	30750
XopQ	HopQ1, RipB	-	-	14830	-
XopR	-	02980	-	41450	03860
XopX1	HopAE1	05660	05860	38620	-
XopX2	HopAE1	05670	05870	38610	-
XopZ1	HopAS1	21660	36240	30390	-
XopAC	AvrAC	16640	16480	-	17460
XopAD	RSc3401	-	-	12990	31140
XopAE	HpaF/G, PopC	-	02610	33570	-
XopAG	AvrGf1, HopAG	-	-	38380	-
XopAL1	Eop3	31840	31680	12630	30770
XopAL2	Eop3	17770	17290	27190	-
XopAM	HopR1	33630	29860	14610	-
XopAR	-	-	04830	39670	05760
XopAT	-	-	-	12980	31130
Other type III-secreted proteins					
HrpW	PopW	32140	31380	12930	31060
XopA	Hpa1	31930	31590	12720	30850
HpaA	-	32090	31430	12880	31010

^a^Locus tag number prefix: XCCFBP5825_m001

^b^Locus tag number prefix: XCICFBP1606_m001

^c^Locus tag number prefix: XCICFBP2527_m001

^d^Locus tag number prefix: XCRCFBP5828_m001

^e^not detected

**Fig. 3:**
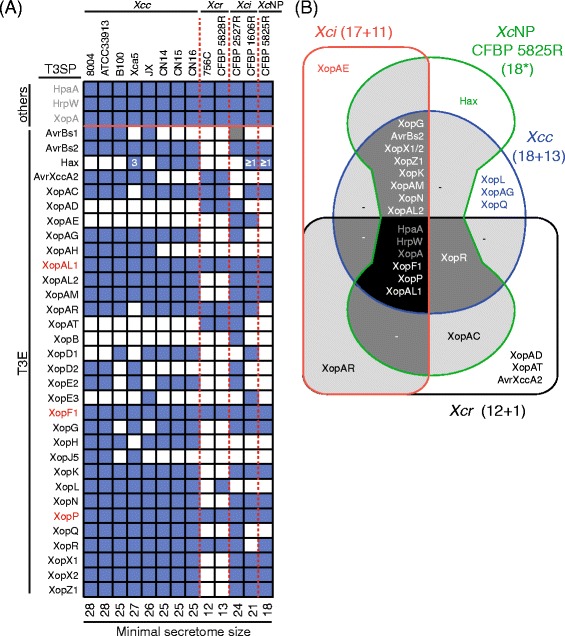
Diversity of type III secretome in *Xanthomonas campestris*. **a** Presence (blue)/absence (white) matrix of genes encoding putative T3SP including T3E (black font) and other accessory proteins (others; grey font) was searched in genomic sequences by tblastn using known T3SP protein sequences (http://www.xanthomonas.org/t3e.html). *X. campestris* core T3E are indicated in red. Grey and blue boxes correspond to protein identities ranging from 40-60 % and 60-100 % over the entire length of the reference protein. Numbers indicate copy numbers of *hax* genes. The minimal size of the secretome is indicated below the table. **b** Venn diagram illustrating the overlap between the core secretomes of *X. campestris* pathovars: *campestris* (*Xcc*), non-pathogenic (*Xc*NP), *incanae* (*Xci*) and *raphani* (*Xcr*). Numbers in brackets indicate the number of the core and accessory type III secreted proteins as inferred from published genome sequences in those pathovars. Genes orthologous to known type III secreted proteins (www.xanthomonas.org) were identified by Blast. Asterisk indicates that all predicted type III secreted proteins of strain CFBP 5825R were used in this analysis. *Xcc*: *X. campestris* pv. *campestris*, *Xci*: *X. campestris* pv. *incanae, Xcr*: *X. campestris* pv. *raphani*, *Xc*NP: *X. campestris* non-pathogenic

The core type III secretomes were determined for each pathovar and compared with each other. Sizes of the core secretomes ranged from 12 for pathovar *raphani* to 18 for pathovar *campestris* (Fig. 3). Only six T3SP were conserved among all the 13 *X. campestris* strains. *hrpW*, *xopA* and *hpaA* put aside, only *xopF1*, *xopP* and *xopAL1* T3E genes were detected in all genomes (Fig. 3a). The type III secretome of pathovar *raphani* contained three T3SP (XopAD, XopAT and AvrXccA2) absent from the core secretomes of the other pathovars and was very different from the T3SP repertoire found in the other three pathovars. Strain belonging to pathovars *campestris*, *incanae* and NP shared 15 T3SP out of 17/18 present in their core secretomes. These results suggest that breeding of disease resistance should focus on the *in planta* recognition of the core T3E XopF1, XopP and XopAL1 in order to achieve broad-spectrum resistance in *Brassicaceae* against most *X. campestris* strains.

### Analysis of the transcriptome of strain CFBP 5828R by RNA sequencing improves genome annotation

In order to identify genes co-regulated with the T3S system, strain CFBP 5828R of *X. campestris* pathovar *raphani* was transformed either with an empty vector (pBBR1MCS-2) or the same vector expressing a constitutively active form of the *hrp* master regulator HrpG (HrpG*, mutation E44K). Total RNA was purified from cells growing exponentially in MOKA rich medium and derived from three independent biological experiments. After removal of rRNA by oligonucleotide-capture, these six samples were size-fractionated to discard the smallest RNA fraction (less than 200bp) and were subjected to strand-specific RNA sequencing on an Illumina HiSeq2000 platform. Sequencing yielded from 12,151,406 to 23,835,557 reads of 51 bp per sample containing 3–16 % of reads corresponding to rRNAs plus tmRNAs [Genbank Sequence Read Archive: SRR1025987 to SRR1025992, http://www.ncbi.nlm.nih.gov/Traces/sra/?study=SRP032762].

The CFBP 5828R genome sequence was first re-annotated taking advantage of this large dataset using Eugene-P software [37]. Expression of all but 28 protein-coding genes (0.6 %) could be detected by RNA sequencing. Out of the 130 ncRNAs obtained by *in silico* ncRNA prediction, 35 (27 %) were not supported by RNA sequencing suggesting that these may either be artifacts or only expressed in specific conditions. This new annotation based on RNA sequencing includes 17 new coding sequences (Table 2). Based on these expression data, 1474 transcriptional starts could be determined leading to the reannotation of 95 translational start sites. One hundred forty-five new ncRNA could also be annotated. Several ncRNA have recently been shown to be important for *Xanthomonas* pathogenicity [38, 39]. Among the 23 ncRNA identified in *X. euvesicatoria* strain 85–10, twelve ncRNA (sX1, sX2, sX5, sX7, sX10-14, 6S, asX1 and asX4) are detected in all *X. campestris* genomes analyzed in this study suggesting a biological significance [38]. Expression of all those ncRNA could be detected in *X. campestris* pv. *raphani* strain CFBP 5828R though only three reads were identified for sX5 (Additional file 1).

In conclusion, the structural annotation of protein-coding genes and ncRNA was significantly improved by the use of RNA sequencing.

### Transcriptomic analysis of CFBP 5828R by RNA sequencing identifies the *hrpG* regulon

**Fig. 4:**
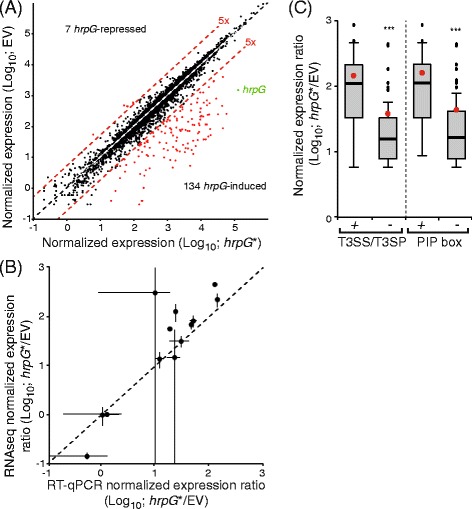
Identification of the *hrpG* regulon of strain CFBP 5828R of *X. campestris* pv. *raphani* by RNA sequencing. **a** Dot plot of the normalized expression levels of all genes in presence of hrpG* versus the empty vector (EV). The diagonal indicates genes which expression level is identical in both conditions. Red lines indicate boundaries where gene expression is induced five-fold relative to the other condition. Red dots indicate genes which expression is significantly induced at least five fold relative to the other condition. The *hrpG* gene is indicated in green. **b** Dot plot of the expression ratios (hrpG* versus EV) of 13 genes determined either by RNA sequencing (RNAseq) or quantitative RT-PCR (RT-qPCR). The diagonal indicates genes which expression ratios are identical with both techniques. Bars indicate standard deviations. **c** Box plot of the normalized expression ratios of *hrpG*-induced genes encoding the T3S system (T3SS) and type III secreted proteins (T3SP) versus the other genes (−). Box plot of the normalized expression ratios of *hrpG*-induced genes with a PIP box (TTCGB-N_15_-TTCGB) in their promoter region versus the other genes (−). Red dots indicate the mean values. Significant mean difference was detected using a Welch-test (***, *P*-value < 0.001)

In order to identify *hrpG*-regulated genes, RNA sequencing reads from the individual libraries (hrpG* or empty vector) were mapped to the re-annotated genome sequence of strain CFBP 5828R. Read counts per objects were used to calculate differential gene expression with *R* (v2.13.0) and DESeq package (v1.4.1) [40]. This approach identified 134 and seven genes the expression of which was induced and repressed more than five fold (*p* < 0,001), respectively, in the strain ectopically expressing hrpG* when compared to the strain containing the empty vector (Fig. 4a, Table 4, Additional file 1). Notably, biological reproducibility was extremely good though slightly more biological variability was observed among the empty vector controls (Additional file 2). To validate these data, RT-qPCR experiments were performed for 13 genes including constitutively expressed, induced and repressed genes (Fig. 4b). A positive correlation was observed between RNA sequencing and RT-qPCR results thus validating the RNA sequencing approach with an independent method. Not so surprisingly, the dynamic range of RT-qPCR was narrower than that of RNA deep-sequencing.

**Table 4:** Properties of *hrpG*-regulated genes in *X. campestris* pv *raphani* strain CFBP 5828R grown in MOKA medium

Locus Tag^*a*^	Gene names	Fold change (hrpG*/EV)^b^	Product	PIP box^c^	Conservation in other *Xc* ^d^
Genes of the *hrp* gene cluster and encoding putative type III secreted proteins					
3860	*xopR*	14	*Xanthomonas* outer protein R	yes	13
5760	*xopAR*	220	*Xanthomonas* outer protein AR	-	12
17460	*xopAC*	127	*Xanthomonas* outer protein AC	-	12
19470	*avrXccA2*	34	AvrXccA2	-	7
30660	*-*	16	hypothetical protein	-	12
30670	*-*	53	hypothetical protein	-	12
30680	*-*	8	hypothetical protein	-	12
30720	*bgl1*	6	beta-glucosidase	-	12
30730	*-*	473	ncRNA	yes	13
30740	*-*	80	hypothetical protein	-	13
30750	*xopP*	68	*Xanthomonas* outer protein P	yes	13
30760	*-*	6	ncRNA	-	13
30770	*xopAL1*	359	*Xanthomonas* outer protein AL1	yes	13
30780	*-*	221	hypothetical protein	(+)	8
30790	*-*	298	hypothetical protein	(+)	13
30800	*-*	222	hypothetical protein	(+)	13
30810	*-*	60	hypothetical protein	(+)	11
30820	*-*	66	hypothetical protein	(+)	13
30840	*hpa2*	885	type III secretion-system related transglycosylase	yes	13
30850	*hpa1/xopA*	504	Hpa1, *Xanthomonas* outer protein A	yes	13
30860	*hrcC*	81	HrcC protein	-	13
30870	*hrcT*	142	HrcT protein	(+)	13
30880	*hrpB7*	67	HrpB7 protein	(+)	13
30890	*hrcN*	129	type III secretion system ATPase	(+)	13
30900	*-*	217	type III secretion system protein HrpB	(+)	13
30910	*hrpB4*	168	type III secretion system protein	(+)	13
30920	*hrcJ*	218	HrcJ protein	(+)	13
30930	*hrpB2*	303	HrpB2 protein	(+)	13
30940	*hrpB1*	307	HrpB1 protein	yes	13
30950	*hrcU*	113	type III secretion system protein HrcU	yes	13
30960	*hrcV*	63	type III secretory pathway component	(+)	13
30970	*hpaC*	116	HpaC protein	(+)	13
30980	*hrcQ*	133	HrcQ protein	yes	13
30990	*-*	208	type III secretion system protein	(+)	13
31000	*-*	147	hypothetical protein	(+)	13
31010	*hpaA*	173	HpaA protein	(+)	13
31020	*hrcD*	356	HrcD protein	yes	13
31030	*hrpD6*	304	HrpD6 protein	(+)	13
31040	*hrpE*	345	type III secretion system pilus protein	(+)	13
31050	*hpaB*	454	type III secretion system export control protein	(+)	13
31060	*hrpW*	128	glycosylhydrolase-like type III secretion system protein	(+)	13
31070	*xopF1*	70	hypothetical protein = XopF1	(+)	13
31080	*hpa3*	148	Hpa3 protein, type III secretion system	yes	5
31090	*-*	32		yes	3
31100	*-*	12	ncRNA	-	3
31110	*-*	79	AutoIPR: IPR008490:Transposase InsH, N-terminal;	(+)	3
31120	*-*	14	ncRNA	(+)	3
31130	*xopAT*	11	*Xanthomonas* outer protein AT	yes	3
31140	*xopAD*	25	Shikimate kinase, *Xanthomonas* outer protein AD	-	5
31160	*-*	34		-	5
31170	*hrpF*	66	type III secretion system host membrane insertion protein	-	13
31180	*-*	7		-	10
31690	*hrpX*	35	AraC family transcriptional regulator = hrpX	-	13
31720	*hrpG*	89	transcriptional regulator HrpG	-	13
31730	*-*	7	ncRNA	-	13
42660	*xopL*	45	*Xanthomonas* outer protein L	yes	9
Other genes					
430	*-*	10	methyltransferase	yes	13
1770	*dctP*	6	dicarboxylate transport system, periplasmic substate binding component	-	13
1780	*dctQ*	6	dicarboxylate transport system, small permease component	-	13
1790	*dctM*	6	dicarboxylate transport system, large permease component	-	13
1800	*xylB3*	6	xylan 1,4-beta-xylosidase/alpha-N-arabinofuranosidase	-	13
4520	*-*	20	transcrictional regulator, MarR family	-	13
4530	*-*	20	hypothetical protein	-	11
4540	*-*	15	hypothetical protein	-	12
4550	*tsr2*	9	membrane-anchored chemotaxis sensory transducer	-	12
4870	*pcaK*	30	4-hydroxybenzoate/protocatachuate MFS transporter	-	13
4880	*-*	6	hypothetical protein	-	13
5010	*-*	8	oxygenase subunit	-	13
5020	*-*	12	oxygenase subunit	-	13
5530	*-*	13	hypothetical protein	-	13
5540	*-*	18		-	13
5660	*virK*	32	hypothetical protein	-	13
5770	*-*	49		-	12
5790	*-*	44	AutoIPR: IPR008490:Transposase InsH, N-terminal;	-	1
5800	*-*	43		-	11
5810	*-*	33		-	11
6120	*-*	8	hypothetical protein	-	13
8610	*pglA1*	90	polygalacturonase	yes	13
9460	*celS2*	10	cellulase/xyloglucan hydrolase	yes	10
9760	*-*	28	hypothetical protein	-	8
9930	*leuC*	8	isopropylmalate isomerase large subunit	-	13
9940	*leuD*	9	isopropylmalate isomerase small subunit	-	13
9950	*ubiE2*	8	ubiquinone/menaquinone biosynthesis methyltransferase	-	13
9960	*leuB*	8	3-isopropylmalate dehydrogenase	-	13
11650	*-*	20		-	1
12120	*-*	13	hypothetical protein	-	3
12940	*-*	55	hypothetical protein	-	13
13330	*-*	59	hypothetical protein	-	13
13340	*-*	431		-	13
13350	*-*	188	hypothetical protein	-	10
13360	*-*	450	hypothetical protein	-	13
13400	*-*	24	secreted lipase	yes	13
14380	*-*	7	ncRNA	-	13
14820	*-*	0,11	Endoproteinase Arg-C (C-terminal fragment)	-	13
16210	*-*	9	cysteine protease	yes	7
16450	*-*	8	serine endopeptidase	-	13
16460	*-*	19	hypothetical protein	-	13
16470	*-*	99	serine endopeptidase	-	13
16480	*-*	41	ncRNA	-	13
17350	*-*	56	Metallopeptidase	-	13
19460	*-*	29	peptidase	-	13
19650	*-*	14	hypothetical protein	-	13
20940	*pglA2*	14	polygalacturonase	yes	13
20950	*-*	30	putative pectate lyase	(+)	1
20960	*-*	54	putative Serine/cysteine peptidase protein	yes	1
20970	*-*	10	AutoIPR: IPR000070:Pectinesterase, catalytic; IPR011050:Pectin lyase fold/virulence factor;	-	1
23600	*-*	8	hypothetical protein	-	13
23610	*-*	116	Metallopeptidase	-	13
26170	*-*	31	TonB-dependent outer membrane receptor precursor	yes	13
26550	*-*	46	MFS glucose importer	-	13
26750	*-*	16	hypothetical protein	-	13
26760	*rpoE4*	8	RNA polymerase ECF-type sigma factor	-	13
26770	*-*	7	hypothetical protein	-	13
26780	*-*	8	Subtilase family serine protease	-	13
26790	*-*	7	ABC transporter heme permease CcmC	-	13
26810	*-*	6	cytochrome c-type biogenesis protein CcmE	-	13
26820	*-*	6	C-type cytochrome biogenesis membrane protein CcmF	-	13
26830	*-*	8	Thiol:disulfide interchange protein (c-type cytochrome biogenesis protein CcmG)	-	13
26840	*-*	5	Formate-dependent nitrite reductase complex nrfFG subunit precursor.	-	13
27610	*-*	15	hypothetical protein	-	13
28890	*cah2*	9	carbonate dehydratase	-	13
28970	*-*	21	transcriptional regulator, MarR family	-	13
29010	*-*	6	hypothetical protein	-	13
29100	*-*	0,16	Major facilitator superfamily protein	-	13
29120	*oprN5*	0,14	outer membrane efflux protein	-	13
29130	*-*	0,19	transcriptional regulator, MarR family	-	13
29740	*-*	7	TonB-dependent outer membrane receptor precursor	-	13
32520	*-*	56	hypothetical protein	-	13
32540	*-*	53	ncRNA	-	13
33090	*-*	9	hypothetical protein	-	13
33100	*-*	11	hypothetical protein	-	13
35670	*-*	13	transcriptional regulator, LysR family	(+)	13
35680	*ligA*	34	protocatechuate 4,5-dioxygenase subunit alpha	(+)	13
35690	*ligB*	54	protocatechuate 4,5-dioxygenase subunit beta	yes	13
37110	*-*	0,11	hypothetical protein	-	13
37120	*-*	0,18	hypothetical protein	-	13
37610	*pel3*	0,14	pectate lyase	-	13
40980	*-*	30	disulfide-isomerase	-	13
42070	*-*	34	Acid phosphatase precursor	-	13
42650	*-*	7	integrase	-	12
45310	*-*	20	hypothetical protein	-	12

^a^Locus tag number prefix: XCRCFBP5828_m001

^b^Only genes with both a fold change higher than five and an adjusted *P*-value lower than 0.001 are shown. *EV* empty vector

^c^yes: Presence of PIP box (TTCGB-N_15_-TTCGB) in the proper orientation in front of the gene. (+): Presence of a PIP-box consensus in the proper orientation in front of a possible operon

^d^Numbers indicate how many of the 13 *Xc* genomes contain at least one homologue of the considered genes as determined by OrthoMCL for protein-coding genes and blastn for ncRNAs

The vast majority of genes with a *hrpG*-dependent expression are positively regulated by *hrpG* (134 out of 141). As expected, genes encoding the Hrp T3S system are included in this list as well as all genes coding for predicted T3SP (56 out of 134). Interestingly, genes of the T3S system and its T3SP are the most highly induced suggesting that T3SP candidates should be expected among genes with the highest induction ratios (Fig. 4c). In *Xanthomonas*, *hrpG*-dependent expression depends in a large part on the HrpX regulator which activates promoters containing the PIP box. PIP boxes are found in front of most operons encoding the T3S system and T3SP (39 out of 56; 70 %). In contrast, only 16 % of the 78 other genes also have a PIP motif in their promoter region. HrpG-dependent induction of gene expression without a PIP box is globally less intense (Fig. 4c) suggesting that *hrpX*, as in other *Xanthomonas,* is also a major regulator of the *hrpG* regulon in *X. campestris* pv. *raphani* strain CFBP 5828R. Besides “protein secretion” mediated by the T3S system, the remaining *hrpG*-induced genes are significantly enriched in the following GO terms (Additional file 3): cytochrome complex assembly (GO:0017004); branched-chain amino acid biosynthetic process (GO:0009082); respiratory chain complex IV assembly (GO:0008535); heme transport (GO:0015886; GO:0015232) and proteolysis and peptidase activity (GO:0006508; GO:0070011). Because extracellular protease activity was shown to be important for pathogenicity in *X. campestris* pv. *campestris* strain 8004 [41] and to be dependent on *hrpG* in *X. euvesicatoria* strain 85–10 [13], we measured the global extracellular protease activity of strain CFBP 5828R, strains carrying hrpG* or the empty vector (Additional file 4). To this end, strains were spotted and grown on MOKA medium containing milk proteins. Surprisingly, the strain carrying hrpG* showed a reduced degradation of milk proteins compared to strains without hrpG*. HrpG does repress the expression of the *arg-C* endoprotease gene XCRCFBP 5828_m00114820 (nine-fold repression in the hrpG* strain, Table 4). Yet, its basal expression levels are low compared to the eight protease genes the expression of which is induced in the hrpG* strain (XCRCFBP 5828_m00116210, XCRCFBP 5828_m00116450, XCRCFBP 5828_m00116470, XCRCFBP 5828_m00117350, XCRCFBP 5828_m00119460, XCRCFBP 5828_m00120960, XCRCFBP 5828_m00123610, XCRCFBP 5828_m00126780; Additional file 1). These results suggest that extracellular protease activity is regulated post transcriptionally by *hrpG*.

In contrast to *X. euvesicatoria* strain 85–10, the expression of ncRNA sX5, sX11 and sX12 was not *hrpG*-dependent in *X. campestris* pv. *raphani* strain 5828R [38]. Yet, the expression of eight novel ncRNA was positively regulated by *hrpG* (*p* < 0.001) (Table 4). Four ncRNA are encoded within the *hrp* gene cluster. Among the eight *hrpG*-regulated ncRNA, three are antisense RNA to the T3E genes *xopR, xopL* and *xopP* and one is antisense to the regulatory gene *hrpG*. The biological functions of those ncRNAs remain to be determined experimentally in *X. campestris*.

As for the seven repressed genes of the *hrpG* regulon, these encode an endoproteinase (locus tag 14820), the Pel3 pectate lyase (37610), a small gene cluster comprising two putative transporters (29100 and 29120) and one transcriptional regulator of the MarR family (29130) and two clustered hypothetical proteins (Table 4). With such a limited number of genes, no significant enrichment in gene ontology (GO) terms could be identified among the *hrpG*-repressed genes.

## Discussion

### Genomic diversity in the *X. campestris* species

The 13 *X. campestris* genome sequences now available provide a panorama of the four major genomic groups composing this species. With a complete T3S system and 19 predicted T3E, it may not be appropriate to consider strain CFBP 5825 to be non-pathogenic as proposed [4]. The NP designation only indicates that none of the *Brassicaceae* tested at the time were appropriate hosts for these strains under the inoculation conditions tested. Future studies should determine if strain CFBP 5825 possesses for instance a functional T3S system and if it is virulent on at least one *Brassicaceae* plant.

A great genomic diversity can be observed both at the intra- and inter-pathovar levels. Previous studies already demonstrated that the pathovar *campestris* is composed of at least three clades [32]. This study suggests that comparable diversity is expected for pathovars *raphani* and *incanae*. The lack of common CRISPR spacers between the two strains of the pathovar *raphani* is particularly striking. Yet, a polyphyletic origin of this pathovar is unlikely since the CRISPR repeats are identical between the two strains. Thus, it suggests that the two strains diverged a long time ago to allow the loss of all ancestral spacers in at least one strain and the likely acquisition of a significant number of new spacers since this event. Importantly, no CRISPR loci could be identified in the other strains so that genomic diversity within this species cannot be precisely determined using this tool and should thus rely on MLSA [4], AFLP [32] or MLVA (multilocus variable number of tandem repeats analysis) schemes as developed for other *Xanthomonas* species [42–44].

It is tempting to compare our results to *P. syringae* where a similar analysis was conducted [45]: the *P. syringae* core genome is composed of 3397 genes (genomes comprise from ca. 5000 to 8000 genes) which is close to the 3481 genes for *X. campestris.* In both instances, comparable numbers of strains were analyzed: 19 *P. syringae* genomes vs. 13 for *X. campestris*. While estimation of core genomes is rather insensitive to annotation quality or homogeneity, the pan genome size can be drastically affected. This could be one of the explanations for the rather small pan genome of the 13 *X. campestris* strains (6472 genes) compared to the 12,749 genes for the 19 *P. syringae* strains. Thus, *X. campestris* appears as a rather homogeneous genomic group compared to *P. syringae* which is considered as composed of several genospecies [46]. These observations are in agreement with the fact that *P. syringae* species has a larger host range compared to *X. campestris* that only infect plant of the *Brassicaceae* family.

### *X. campestris* core type III effectome is reduced to three genes

Considering the high quality of the genomes obtained in the frame of this analysis, one can be rather confident in the predicted sizes of *X. campestris* type III secretomes. Pan type III secretomes range from 13 to 31 for pathovars *raphani* and *campestris* respectively (Fig. 3b). Despite having a large core genome, the predicted core effectome of the 13 *X. campestris* strains was found to consist of only three bona fide effectors (XopF1, XopP and XopAL1) plus three T3SP (XopA, HrpW and HpaA) which are likely to be involved in the type III secretion and translocation process itself. The core effectomes of 138 *X. axonopodis* strains and 65 *X. axonopodis* pv. *manihotis* strains are made of ca. eight and six candidate T3Es, respectively [47, 48]. Yet, these two effectomes only have XopN in common. Combined with our results, only XopF1 may still be considered as a core *Xanthomonas* effector though pseudogenized in many *X. axonopodis* strains [47]. This situation is also reminiscent of type III effectome studies performed in *P. syringae* and *R. solanacearum*. In the species complex *R. solanacearum*, 22 out of the 94 T3E composing the pan effectome are conserved in all eleven strains analyzed [49]. The *P. syringae* core effectome is limited to five T3E (HopM, AvrE, HopAA, HopAH and HopI) though none is strictly conserved and intact in all 19 strains [45]. Therefore, there is no overlap between *P. syringae* and *X. campestris* core effectomes. These results challenge the concept of core effectomes as soon as increased biodiversity is analyzed at the species level or above. It also indicates that no universal set of effectors is used to infect plants, which could suggest that pathogenic bacteria use host-specific strategies to circumvent plant immunity or promote susceptibility. Yet, the functional redundancy observed within effectomes rather suggests that bacteria may use a repertoire of unrelated effectors to target conserved plant targets. This later hypothesis is supported by the identification of important plant susceptibility hubs such as RIN4, SWEETs or RLCK VII (For review, [50]). To date, the molecular functions of XopF1, XopP T3E remain unknown. As for XopP, it was recently shown to block peptidoglycan- and chitin-triggered immunity in rice by inhibiting the U-box ubiquitin ligase OsPUB44, a positive regulator of basal immunity [51].

### RNA sequencing refines the annotation of *X. campestris* pv. *raphani* strain CFBP 5828R T3E genes and ncRNA

RNA sequencing approaches in plant pathogens including *Xanthomonas* are still in their early days and were so far used to identify regulons, transcriptional start sites or ncRNAs [38, 52–54]. In this study, the sequencing of the transcriptome of *X. campestris* pv. *raphani* strain CFBP 5828R produced ca. 111 million reads of 51-bp resulting in 5.66 Gb of raw data. The use of a custom *Xanthomonas*-optimized oligonucleotides set allowed a high ribodepletion efficacy (2–13% rRNA/tRNA/tmRNA reads) so that more than 93% of the total reads were specifically mapped to mRNAs. 1000-fold coverage of the genome was achieved which is comparable to studies in *X. campestris* pv. *campestris* [54] but significantly higher than studies in *X. euvesicatoria*, *X. citri* pv. *citri* and *X. campestris* pv. *campestris* (10-fold, 700-fold and 400-fold respectively) [38, 52, 53]. The size of this dataset is also far above the accepted limit (5–10 millions non-rRNA reads per library) for correct expression profiling, gene discovery or gene reannotation [55]. The RNA reads were first used to improve the structural annotation of the genome using the Eugene-P pipeline. One important difference of our RNA sequencing approach with most of the above-mentioned reports is the use of strand-specific libraries. Such libraries enable to assign individual reads to a specific DNA strand in the genome and therefore allow, for instance, to identify overlapping or antisense RNA molecules: 72 ncRNA overlap with CDS such as the three T3E genes *xopR, xopL* and *xopP* in *X. campestris* pv. *raphani* strain CFBP 5828R. Early functional studies in *X. euvesicatoria* have shown that several ncRNA contribute to pathogenicity [38, 39]. Only few new mRNA were identified. Yet, the major improvement is a better characterization of transcriptional start sites (TSS). This latter point is particularly relevant for T3E genes which are often characterized by an atypical codon usage and a lack of homology with known genes, thus preventing proper annotation of their TSS (e.g. [56]). As an illustration, three T3E proteins are likely to be longer than automatically annotated. Based on their 5’-UTR, *xopAD*, *xopAL1* and *xopAT* may encode N-terminal extensions of 169, 9 and 228 amino acids, respectively. Compared to the published annotation of *X. campestris* pv. *raphani* strain 756C [21], XopAT and XopAD may be 21 and 57 amino acids shorter in strain CFBP 5828R. For XopAL1, our data support the annotation of *X. campestris* pv. *campestris* strain B100 which is 27 amino acids longer than in strain 8004.

### The *hrpG* regulon in *X. campestris* pv. *raphani* strain CFBP 5828R encompasses all predicted T3E, T3SP and T3S system genes

The small effectome of *X. campestris* pv. *raphani* suggests that more effectors could be discovered. To date, the most productive strategy remains to determine genes that are co-regulated with the genes encoding the Hrp type III secretion apparatus [13, 26, 27]. In our study, we chose to determine the *hrpG* regulon by RNA sequencing using a constitutive active form of this regulator HrpG*. RNA sequencing has a higher dynamic range than micro-arrays and also offers full genome coverage. The use of the hrpG* mutant allele was previously used successfully [13] and permits the growth of all bacterial strains in a single medium thus minimizing the noise due to metabolic responses unrelated to the *hrp* gene regulation. For instance, RNA sequencing of *X. campestris* pv. *campestris* grown in synthetic *hrp* gene-inducing medium MMX vs. rich medium resulted in the identification of a regulon of more than 600 genes mostly involved in bacterial metabolic adaptation [52]. In addition, increased expression in MMX was observed in only five out of 12 T3E genes resulting in poor predictive potential for T3E gene discovery [52].

Comparing the size and composition of *hrp* regulons is difficult because it depends on the biological system, the experimental design, the statistical analyses and the chosen cut-off values. For *X. campestris* pv. *raphani*, we intentionally selected stringent values (>5 fold induction/repression and *p* < 0,001) so that the resulting regulon is limited to 141 genes (3 % of the genes), 95 % of which are induced. This regulon size is comparable to the reported *R. solanacearum hrpB* regulon [26] and the *P. syringae hrpL* regulon [57] but smaller than the regulon determined in *X. campestris* pv. *campestris* grown in synthetic *hrp* gene-inducing medium MMX [52]. In these two later examples, only 80 % of the genes were positively co-regulated with the T3S system genes. Genes of the *hrpG* regulon are well conserved in *X. campestris* since 74% of those are detected in the 13 genomes inspected (Table 4). Importantly, all known T3E, T3SP and T3S system genes were found to belong to the *X. campestris* pv. *raphani hrpG* regulon. For instance, 34-fold induction of *avrXccA2* expression in *X. campestris* pv. *raphani* strain CFBP 5828R upon hrpG* expression (Table 4) provides the first experimental hint for AvrXccA2 being a T3E candidate. One expects to find unknown T3E genes among the genes which expression is highly upregulated by HrpG (13 genes with induction fold higher than 50, Table 4) and among those with PIP promoter motifs (12 genes, Table 4). This repertoire of 22 *hrpG*-induced genes, once processed with T3E prediction tools [36, 58], constitutes a manageable list to mine experimentally for novel type III effectors in *X. campestris* pv. *raphani*.

## Conclusions

A deep knowledge of the genomic diversity of *X. campestris* is needed to develop effective molecular typing schemes. This study presents a first genomic coverage of the pathovars of *X. campestris*. Core- and pathovar-specific proteomes were determined as well as the repertoire of Xop effector proteins that are used by bacteria to subvert plant immunity. Using RNA sequencing, a set of genes co-regulated with the T3S system including non-coding RNAs was identified which should contribute to our understanding of the virulence strategies of this important species of phytopathogens.

## Methods

### Bacterial strains, plasmids and growth conditions

Strains and plasmids used in this study are listed in Table 1. *X. campestris* strains were grown at 28 °C in MOKA medium [59]. *Escherichia coli* cells were grown on LB (lysogeny broth) medium at 37 °C. For solid media, agar was added at a final concentration of 1.5 % (w/v). Antibiotics were used at the following concentrations: 50 μg/ml kanamycin, 50 μg/ml rifampicin, spectinomycin 40 μg/ml. To select for spontaneous rifampicin-resistant *X. campestris* mutants, overnight cultures in liquid MOKA were plated on MOKA-Rif medium at high density. The CFBP strains are available from the CIRM-CFBP collection of plant-associated bacteria (Angers, France, http://www6.inra.fr/cirm_eng/CFBP-Plant-Associated-Bacteria).

### Genome sequencing and assembly

Shotgun sequencing of genomic DNA was performed on HiSeq2000 Illumina platform. Paired-end (ca. 300-bp inserts) and mate-pair (ca. 3-kb inserts) libraries were sequenced to generate 101-bp and 51-bp paired reads respectively. Genome assembly was performed using a combination of SOAPdenovo (Short Oligonucleotide Analysis Package, version 2.04) [30], SOAPGapCloser (version 1.12) and Velvet (version 1.2.02) [31] assemblers. Contigs obtained were re-ordered into pseudomolecules (corresponding to chromosome or plasmids) with the Mauve software (version 2.3.1 build 1) [60] based on the reference genome *X. campestris pv. campestris* strain 8004, and public *Xanthomonas* plasmid sequences available on NCBI (*X. axonopodis pv. citri* str. 306 plasmid pXAC64, NC_003922; *X. axonopodis pv. citri* str. 306 plasmid pXAC33, NC_003921; *X. euvesicatoria* str. 85–10 plasmid pXCV183, NC_007507; *X. euvesicatoria* str. 85–10 plasmid pXCV38, NC_007506; *X. euvesicatoria* str. 85–10 plasmid pXCV19, NC_007505; *X. euvesicatoria* str. 85–10 plasmid pXCV2, NC_007504; *X. euvesicatoria* plasmid pXV64, NC_004987; *X. fuscans* subsp*. fuscans* str. 4834R plasmid pla, NC_022539; *X. fuscans* subsp*. fuscans* str. 4834R plasmid plb, NC_022540; *X. fuscans* subsp*. fuscans* str. 4834R plasmid plc, NC_022542). When necessary, a single contig was split in order to make *dnaA* the first gene in the chromosomal pseudomolecule.

### pBBR-hrpG cloning

hrpG* (E44K) coding sequence was amplified from 8004* strain [61] using oligonuleotides LN635/636 (Additional file 5) and cloned as a *Xho*I/*Hin*dIII DNA fragment into pBBR1-MCS-2 [62]. Plasmids were introduced into *E. coli* by electroporation and into *X. campestris* using pRK2073 as helper plasmid in triparental matings [63, 64].

### RNA extraction, rRNA depletion and pyro-sequencing

For each genotype of *X. campestris* pv. *raphani* strain CFBP 5828R, three independent cultures in MOKA medium were harvested at mid-exponential phase (OD_600nm_ = 0.5) and subjected to RNA extraction as described [37]. After TurboDNAse (Ambion) treatment and quality control using Bioanalyzer RNA6000 Nano kit (Agilent Technologies Genomics), depletion of ribosomal and selected tRNA was performed as described [37] using a custom set of oligonucleotides optimized for the *Xanthomonas* genus (Additional file 5). Single-end RNA sequencing (51-bp reads) was performed on the Illumina HiSeq2000 platform (Fasteris SA, Geneva, Switzerland) as described [37].

### Genome structural annotation

Structural annotation was done using Eugene-P software [37]. This modular software allows the integration of several sources of high-throughput data such as protein similarities, DNA homologies, predicted transcription terminators and transfer RNA genes and others. We trained Eugene-P with the public annotation of *X. campestris* pv. *campestris* strain B100 (available on NCBI website under the accession number NC_010688), *X. campestris* pv. *campestris* strain 8004 (NC_007086) and *X. campestris* pv. *campestris* strain ATCC 33913 (NC_003902) and at a lower weight with the Swissprot database (version of 04 February 2013). For strain CFBP 5828R, all RNA sequencing libraries were merged and used by Eugene-P to predict structural annotations of mRNA as described [37].

### Identification of core and accessory genes in *X. campestris* pathovars

Identification of orthologous groups between genomes was achieved by OrthoMCL analyses (Li *et al.*, [52]) with the 13 genomes. In order to prevent annotation biases during downstream analyses, all published genomes were re-annotated with Eugene-P as described above. OrthoMCL clustering analyses were performed using the following parameters: *p*-value cut-off = 1 × 10^−5^; Percent Identity cut-off = 0; Percent match cut-off = 80; MCL Inflation = 1.5; Maximum weight = 316. We modified OrthoMCL analysis by inactivating the filter query sequence during the BLASTP pre-process. Groups of orthologs corresponding to CDS present in one copy in at least two genomes were extracted from OrthoMCL output files. For the group of strains considered, the core proteome was defined as the OrthoMCL groups represented by a single protein in each strain. The pan proteome was defined as all the OrthoMCL groups present in the group of strains considered plus single copy strain-specific proteins.

### Phylogeny of *X. campestris* genomes

Phylogenetic analysis was performed based on OrthoMCL analyses. Only groups composed of one single protein in each strain was used to build what we defined as the core proteome. For each of these groups, we aligned the protein sequences using MAFFT software [65] and cleaned alignment using trimAl software [66] to remove all positions in the alignment with gaps in 10% or more of the sequences. Alignment files were converted in phylip format. Phylogenetic tree was constructed using PhyML [67].

### Identification of candidate type III-secreted proteins in *X. campestris* strains CFBP 5825R, CFBP 1606R, CFBP 2527R and CFBP 5828R

Protein sequence of T3SP from strains *X. campestris* pv. *campestris* 8004 and B100, *X. euvesicatoria* 85–10, *X. campestris* pv. *raphani* 756C or *X. axonopodis* pv. *citri* 306 (http://www.xanthomonas.org/t3e.html) were used as queries for a *tblastn* analysis on the genomes of the different *X. campestris* strains. T3SP genes were considered as present in the genome sequences when protein alignments shared at least 60% identity over the full length of the reference proteins. Core type III secretomes were defined for each pathovar as proteins present in all sequenced strains of a given pathovar.

### Analysis of RNA sequencing results and statistical analyses

Mapping of RNA sequencing reads on CFBP 5828R genomic sequence was done using the Glint software (Faraut T. and Courcelle E.; http://lipm-bioinfo.toulouse.inra.fr/download/glint/, unpublished) integrated in the Eugene-P pipeline. Parameters used were the following: matches allow no gap, a minimum length of 40 nucleotides and no more than one mismatch. We kept only the reads with the best score when several reads mapped to two different positions with different scores. Reads mapping at two different positions with the same best scores were not considered. Differential expression of genes was calculated with *R* (v2.13.0) using DESeq (v1.4.1) [40]. Variance was estimated using the per-condition argument. *p*-values were adjusted for multiple testing using the Benjamini and Hochberg method [68].

### Quantitative RT-PCR analyses

Two micrograms of total RNA used for the RNA sequencing were subjected to reverse transcription using Transcriptor reverse trancriptase (RT, Roche Applied Science) and 1 μM Random Primer 6 (New England BioLabs). Quantitative PCR was performed using diluted cDNA with 1 μM gene-specific oligonucleotides (Additional file 5) and LightCycler 480 SYBR Green I Master kit on a LightCycler 480 (Roche Applied Science) machine with the following cycling parameters: 9 min 95 °C; 50 cycles of 15 sec 95 °C, 10 sec 60 °C and 10 sec 72 °C. Technical and biological triplicates were performed. Efficiencies of the primer pairs were determined on diluted genomic DNA and were greater than 1.8. Two reference genes expressed constitutively in the RNA sequencing experiment (CFBP 5828R_m00134650 and CFBP 5828R_m00117870) were used for normalization. Expression levels were calculated using the ∆∆Ct method.

### Identification of PIP boxes

The PIP-box motif (TTCGB-N_15_-TTCGB; B represents C, G, or T) was searched in *X. campestris* pv. *raphani* strain CFBP 5828R in JBrowse. Genes with PIP boxes were manually inspected with the following criteria: presence of the PIP-box motif in the sense orientation relative to the transcriptional unit and situated in the 500-bp region upstream of the transcriptional start. Presence of operons was estimated from RNA sequencing data.

### Gene Ontology enrichment studies

Enrichment of specific Gene Ontology terms in gene lists was tested using TOPGO *R* package (v1.14.0) [69].

### Extracellular protease assay

Extracellular protease activity of *Xanthomonas* strains was tested by spotting 10μl of an overnight culture (OD_600nm_ = 0.4) on MOKA plates supplemented with 1 % skimmed milk. Plates were incubated at 28 °C and imaged three days post inoculation.

### Availability of suppporting data

Automated annotation of 13 *X. campestris* genomes and the OrthoMCL analyses performed on the 13 derived proteomes are freely available at the LIPM repository (https://lipm-browsers.toulouse.inra.fr/pub/xanthomix/).

## Additional files

Additional file 1:
**Gene expression levels determined by RNA sequencing in** ***X.*** **campestris** **pv.** ***raphani*** **CFBP 5828R with empty vector or hrpG* plasmid.** (XLSX 835 kb) Additional file 2:
**Scatter plots of the normalized expression levels of the 141** ***hrpG*** **-regulated genes of strain CFBP 5828R of** ***X.*** **campestris** **pv.** ***raphani*** **by RNA sequencing (Table** **4** **, Fig.** **4a** **).** (PDF 635 kb) Additional file 3:
**Enrichment in gene ontology (GO) terms in the** ***hrpG*** **-induced regulon of** ***X. campestris*** **pv.** ***raphani*** **strain CFBP 5828R.** (XLSX 9 kb) Additional file 4:
**Extracellular protease activity of strain CFBP 5828R of** ***X. campestris*** **pv.** ***raphani*** **is repressed by** ***hrpG*.*** (PDF 3290 kb) Additional file 5:
**Oligonucleotides used in this study.** (PDF 865 kb)

## Abbreviations

AFLP: Amplified fragment length polymorphism
CDS: Coding sequence
GO: Gene ontolology
Hrp: Hypersensitive response and pathogenicity
MLSA: Multilocus sequence analysis
MLVA: Multilocus variable number of tandem repeats analysis
ncRNA: non-coding RNA
NP: Non-pathogenic
PAMP: Pathogen-associated molecular pattern
PIP: Plant-inducible promoter
PTI: pattern-triggered immunity
RT: Reverse transcription
RNAseq: RNA sequencing
T3E: Type III effector
T3S: Type III secretion
T3SP: Type III secreted protein
TSS: Transcriptional start site
UTR: Untranslated region
Xop: *Xanthomonas* outer protein
