# Supplementary material for: Genomics and transcriptomics of Xanthomonas campestris species challenge the concept of core type III effectome
Source: BMC Genomics. 2015 Nov 18;16:975. doi: 10.1186/s12864-015-2190-0 (PMC4652430; doi:10.1186/s12864-015-2190-0)
Supplement: Additional file 2: — Scatter plots of the normalized expression levels of the 141 hrpG -regulated genes of strain CFBP 5828R of X. campestris pv. raphani by RNA sequencing (Table 4 , Fig. 4a ). (PDF 635 kb) [file 12864_2015_2190_MOESM2_ESM.pdf]

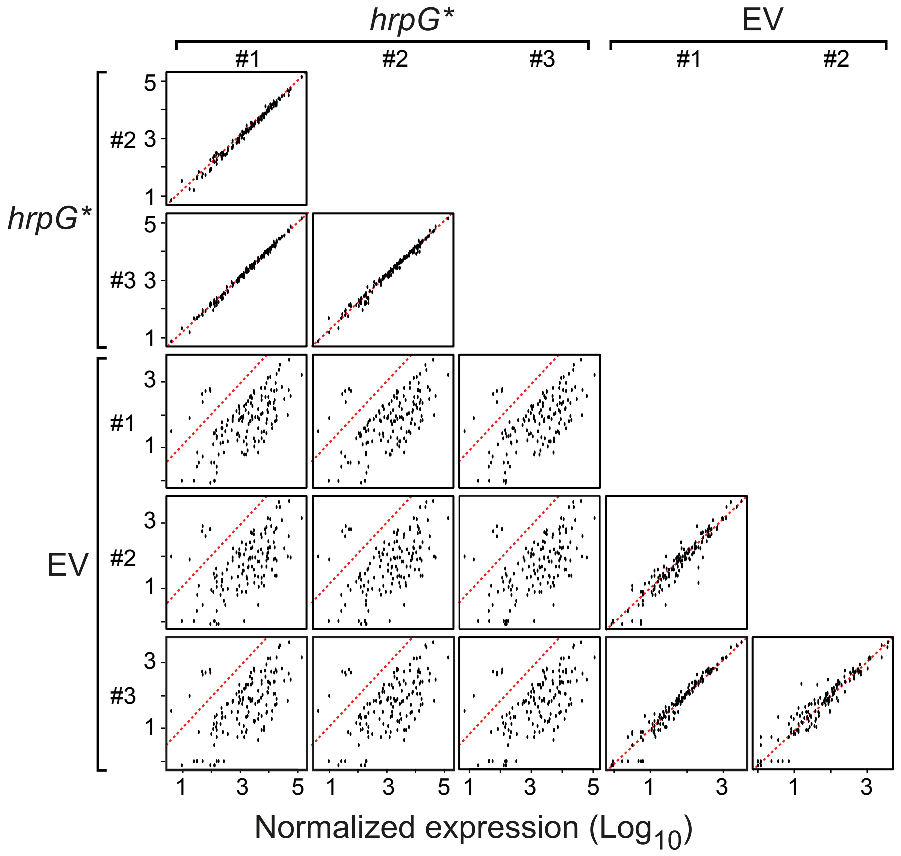

Additional File 2: Scatter plot of the normalized expression levels of the 141 *hrpG*-regulated genes of strain CFBP 5828R of *X. campestris* pv. raphani by RNA sequencing (Table 4, Figure 3A). The different biological samples are compared to assess biological variability and reproducibility. The red diagonals indicate genes which expression levels are identical in both samples.

Roux *et al.*
