# Supplementary material for: Genomics and transcriptomics of Xanthomonas campestris species challenge the concept of core type III effectome
Source: BMC Genomics. 2015 Nov 18;16:975. doi: 10.1186/s12864-015-2190-0 (PMC4652430; doi:10.1186/s12864-015-2190-0)
Supplement: Additional file 4: — Extracellular protease activity of strain CFBP 5828R of X. campestris pv. raphani is repressed by hrpG*. (PDF 3290 kb) [file 12864_2015_2190_MOESM4_ESM.pdf]

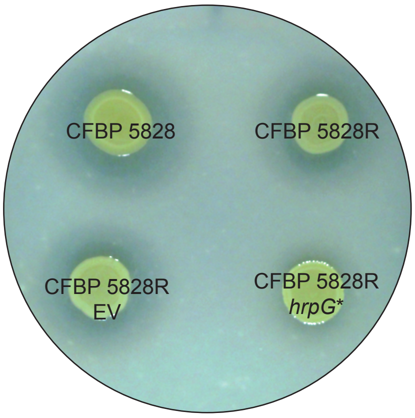

Additional File 4: Extracellular protease activity of strain CFBP 5828R of *X. campestris* pv. *raphani* is repressed by *hrpG.** Bacterial strains were spotted in MOKA plates containing 1% skimmed milk. Haloes formed around the colonies by milk protein degradation are an indirect measure of bacterial extracellular protease activity. Pictures were taken after three days at 28°C.
