# Supplementary material for: Genomics and transcriptomics of Xanthomonas campestris species challenge the concept of core type III effectome
Source: BMC Genomics. 2015 Nov 18;16:975. doi: 10.1186/s12864-015-2190-0 (PMC4652430; doi:10.1186/s12864-015-2190-0)
Supplement: Additional file 5: — Oligonucleotides used in this study. (PDF 865 kb) [file 12864_2015_2190_MOESM5_ESM.pdf]

Additional File 5: Oligonucleotides used in this study

Name	Sequence (5'-3')
for cloning	
LN635	TACTCGAGGGCGTGGGCGCGCGAGTGATG[1]
LN636	TGAAGCTTAGCAAGCTGCGGTGCGATTG
for ribodepletion	
X16S_1101	GACTTAAcCCAAcATcTCAcGAC[2]
X16S_129	ATGTATTcCTCAcCCGTcCGCcA
X16S_1316	CCAATCcGGAcTGAgATAgGGTT
X16S_1447	ACCTGCTTcTGGTGcAAcAAAcT
X16S_1513	ACCTTGTTAcGAcTTCAcCCcAG
X16S_335	CGTGTcTCAGTTcCAGtGTGGcT
X16S_596	ACCAcCAcCTAcGCACGcTTTAC
X16S_64	ACTTGcATGTGtTAGGCcTGCcG
X16S_841	CCCAAcATcCAGTTcGCATcGTT
X23S_1205	CTCAATcCAcCTTcGCAGGcTTA
X23S_125	GGTTTCcCCAtTCGGAtATtGCC
X23S_1467	ATCCAAAcCTAcCGCcTTTcCCC
X23S_1733	TTTcTGCGAcCCTcTTCAGcTAT
X23S_1957	TCGTTAcGcCATTCGTGcAgGTC
X23S_2224	GTCAAAcTAcCCAcCATACAcGG
X23S_2526	CCATAcCcTTGGGAcCGAcTACA
X23S_2744	CGCTTcCCGcTTAgATGcTTTCA
X23S_2836	CATTGcTGTAcTTAcACAcCTGA
X23S_30	AGGcATCcACCGTGTGCGcTtAT
X23S_353	CGTcTTCACTGgAGTGGcCcTTT
X23S_593	TACGgTTAAGcTTGcCAcGAACA
X23S_807	AAATAGcTTTcGAGgAGAAcCAG
X23S_953	TTTTcACGAcGGAcGTTAGcACC
X5S_109	TGAcCTACTcTCGcATGGcTTGA
X5S_57	CACTTcCGaGTTCGGGAtGGgAT
XtRNA_Ala	TTGcAAAGCAgGTGcTCTcCCAGC
XtRNA_Ile	TCTAAcCAcCTGAGcTAcAGACC
for quantitative RT-PCR	
XCRCFBP5828_m00103860_F	CTCTCAGGCTCCCAAGTCAA
XCRCFBP5828_m00103860_R	CGTAACGATGCCAGCGATTT
XCRCFBP5828_m00113360_F	TTCGCCGCATCGATATTC
XCRCFBP5828_m00113360_R	CGTAGCGTGAGGAAGGAATG
XCRCFBP5828_m00117460_F	GTCATTTCTTGGGGGAAAGC
XCRCFBP5828_m00117460_R	CGTGGCAATACCCTCCAG
XCRCFBP5828_m00117870_F	CTACCTCATCGCGATCCAGT
XCRCFBP5828_m00117870_R	AAACCGAGGAACAACATGCC
XCRCFBP5828_m00126170_F	GCAACTACGGAGCGTTCG
XCRCFBP5828_m00126170_R	CGCTGGTTTCTCCCACTG
XCRCFBP5828_m00130750_F	TTGTGGGTTCGTCTGATGTG
XCRCFBP5828_m00130750_R	CAGCCGCGGGTAATAACA
XCRCFBP5828_m00130860_F	TCCATCACCACCTGGATTTC
XCRCFBP5828_m00130860_R	GAGAAACTTCTGCGGAGTGG
XCRCFBP5828_m00130920_F	AGAACGATGCCAACGACAT
XCRCFBP5828_m00130920_R	GCTTTTTCTGCATCCACACC
XCRCFBP5828_m00131030_F	GCAACGATTTCATTGTCTTGC
XCRCFBP5828_m00131030_R	ATGTGCGCGACGATGTCT
XCRCFBP5828_m00131120_F	CCCAGCCAAGAATCCGTTTTATA
XCRCFBP5828_m00131120_R	CTAAGCTCCGAATCAGTGACG
XCRCFBP5828_m00132520_F	ACTACGAGGCAGGACGCTAC
XCRCFBP5828_m00132520_R	TGTATCGTGCTGGACCACTC
XCRCFBP5828_m00134650_F	GGACACCAATAAGACCGTGAA
XCRCFBP5828_m00134650_R	CACATCGTTGAGCTTGTTCAG
XCRCFBP5828_m00137610_F	ATTCCCACCAAGACCACCTT
XCRCFBP5828_m00137610_R	ATGTAGCCTTCGCGGATTT

[1] Underlined bases indicate restriction sites
[2] Lower case bases indicate modified LNA bases
